# Mechanisms and Empirical Modeling of Evaporation from Hardened Surfaces in Urban Areas

**DOI:** 10.3390/ijerph18041790

**Published:** 2021-02-12

**Authors:** Jinjun Zhou, Jiahong Liu, Qi Chu, Hao Wang, Weiwei Shao, Zhuoran Luo, Yongxiang Zhang

**Affiliations:** 1 Faculty of Architecture, Civil and Transportation Engineering, Beijing University of Technology, Beijing 100124, China; zhoujj@bjut.edu.cn (J.Z.); chuqi@bjut.edu.cn (Q.C.); wanghao123612@163.com (H.W.); 15611708278@163.com (Z.L.); 2 State Key Lab of Hydro-Science and Engineering, Tsinghua University, Beijing 100084, China; 3 China Institute of Water Resources and Hydropower Research State Key Laboratory of Simulation and Regulation of Water Cycle in River Basin, Beijing 100038, China; shaoww@iwhr.com

**Keywords:** urban hydrology, evaporation, hardened surfaces, urban environment, urban thermal comfort

## Abstract

Urban evaporation, as an essential part of local water vapor resources in urban areas, has often been underestimated. One possible reason is that the evaporation of urban hardened surfaces is seldom considered and poorly understood in urban evaporation estimation. This study focused on the mechanisms and calculation of evaporation on hardened surfaces in urban areas. Experimental monitoring was used to monitor the processes and characteristics of evaporation on hardened surfaces. Mathematical models based on water quantity constraints were built to calculate evaporation of hardened surfaces. The results showed that: The interception abilities for rainwater and rainfall days of impervious hardened surfaces determine their evaporated water amount, which means no water, no evaporation for the impervious surfaces. The greater evaporation of artificial sprinkling on roads happened in fewer days of rainfall and frost. The evaporation of pervious hardened ground is continuous compared to the impervious surface. Its soil moisture in the sub-layer of permeable concrete decreases periodically with a period of one day. The evaporation of hardened surfaces occupies 16–29% of the total amount of evaporation in the built-up areas in cities. Therefore, the hardened surface evaporation has great significance on the urban hydrological cycle and urban water balance.

## 1. Introduction

Urbanization has led to the expansion of urban population and urban hardened surface area [1,2]. Hardened surfaces refer to the artificial ground or roof structure which are paved with stone, brick, concrete, asphalt and so on. Typical hardened surfaces include asphalt road, cement ground, brick pavement, and building roof, which are the main underlying surfaces in cities. By the end of 2019, the proportion of urban population and hardened surfaces in cities were both above 60% in China [3]. Urban hardened surfaces bring great changes to the urban environment and hydrological process [1,4,5]. The usual results show that the increase of hardened surfaces in cities will reduce the evaporation, increase the sensible heat, increase the surface temperature, and aggravate the effect of urban heat island [6,7,8]. Therefore, the urban hardened surface will change the urban thermal comfort and influence public health [9,10].

Evaporation is one of the main hydrological cycle processes [11,12], and it is also the main way to reduce urban sensible heat and alleviate the urban heat island effect [13]. Urban evaporation refers to the phenomenon of water transformation from liquid to gaseous state in urban areas. Similarly, the evaporation of hardened surfaces refers to the evaporation of water on or under the hardened ground structure. As the main underlying surface in cities, evaporation from hardened surfaces should be an important part of urban evaporation [5,7,14]. Observational data and the Princeton urban canopy model showed that evaporative fluxes from concrete pavements, building rooftops, and asphalt surfaces have a significant impact on the urban surface energy balance [15].

However, the research on evaporation of hardened surfaces is relatively weak, which may be due to two aspects: the monitoring of surface evaporation is difficult and the heterogeneity of hardened surfaces is high [16,17]. Hardened surfaces can be divided into impervious surfaces and pervious surfaces [14,15]. The impervious surface blocks water infiltration and reduces the water availability to be stored in the soil [18]. The water-storage capacity and the maximal wet surface fraction constitute the evaporation area of impervious surfaces [19]. For municipal roads in urban areas, water on roads comes from natural rainfall and artificial sprinkling [17]. Furthermore, the evaporation on the imperious surfaces occurs after rain or after artificial sprinkling [16,20,21,22] and plays a crucial role in road surface temperature simulations on rainy days [23]. Pervious hardened surfaces are structures that connect the surface and underground soil, which mean the rain water can infiltrate into the underground soil and the soil moisture can evaporate into the air through the pervious structure [24]. Yet, the evaporation of hardened surfaces is usually underestimated or even ignored in urban evaporation calculation in existing studies [16,25]. Meanwhile, urban hardened surfaces and human activity altered the land surface radiation balance, energy balance, and water balance [26,27]. Moreover, many parameters used in calculation and simulation, such as the roughness length, albedo, permeability, were changed [23] Less research has been done to revise the parameters of traditional evapotranspiration in urban evaporation calculation. For much research, evaporation is considered to be energy-constrained, and most of the evaporation calculations are based on the energy equation. Meanwhile, few researchers gave a detailed analysis of the hydrological responses of the pervious hard ground [28].

This research mainly focuses on the experimental study of evaporation on impervious and pervious hardened surfaces. The prototype monitoring experiments of artificial sprinklers and natural rainfall were carried out on several impervious ground, pervious, and the natural rainfall monitoring experiments were carried out on the ground of permeable concrete samples. The experiments answer the questions (1) what are the mechanisms that control evaporation from impervious and pervious hardened surfaces, and (2) can evaporation from these surfaces be represented with simple mathematical models? The monitor data are used to validate the model.

## 2. Materials and Methods

Experimental monitoring was carried out for impervious surfaces and pervious hardened surfaces, respectively. The experiment on impervious surfaces is mainly to monitor the water holding depth of the road. Under actual circumstances, the road being uneven or road depression is a popular phenomenon, and the depression area will develop into depression storage after rainfall happens, which will increase the evaporation of the impervious surfaces. In this study, the authors assume that the pavement is flat and not depressed, and the influence of the depression of impervious surfaces is not considered.

For pervious hardened surfaces, the evaporation change should be monitored by measuring soil moisture content of both pervious hardened layer and subsoil layer, which the water can penetrate the pervious hardened structure and the water vapor of subsoil can pass the pervious structure into the air. The evaporation of pervious hardened surface will influence the temperature and humidity of the surface, and the rainfall will directly change the soil moisture in pervious hardened ground, so these elements are also monitored in this study. Soil moisture content is recorded by soil moisture probe, rainfall is recorded by rainfall barrel, RH and temperature are recorded by temperature and humidity self-recording instrument. There are other meteorological factors that affect the evaporation process, such as wind speed, net radiation, and so on [29,30]. These meteorological data are derived from the monitoring data of the weather station.

### 2.1. Experiment on Impervious Ground

The experimental method of intercepting rain water depth is the determination of ground interception capacity under artificial sprinkling. First, the authors chose a piece of clean and flat impervious hardened ground as the monitoring object, and then lay a rectangular plexiglass bottomless tank to adjust the position to ensure that there is no obvious gap between the sides and the ground. Then, they sprinkled the water evenly in the tank with a sprayer until the runoff occurs at a certain point to stop sprinkling water. To measure weight difference of the spray before and after spraying by weighing, which is the amount of water sprayed, the depth of the impervious ground interception rain water is recorded as *H* and the unit is mm. The *H* measured by the above method is calculated as:

(1) $$H=\frac{m}{1000\times A}$$

where, *m* is the amount of sprinkling water at critical runoff in the rainfall area, the unit is g; *A* is the rainfall area of model experiment, the unit is m^2^.

In this study, the size of the tank is 0.5 m × 0.5 m, and the *A* is 0.25 m^2^. The precision of experimental electronic weighing is 1 g and the maximum range is 5 kg. The precision is 0.004 mm in this experiment area, which meets the precision requirement of experimental result 0.01 mm. The most commonly used asphalt pavement, cement pavement, concrete pavement and brick pavement are selected as the experimental monitoring objects of impervious hardened surfaces. Three types of brick pavement were selected for the experiment, and their gap ratios were 2%, 4%, and 6%. Gap ratio is the value of gap area dividing total area in a certain brick pavement area, the bricks sealed with concrete or mortar between them. The number of sprinkling experiments on the same ground is not less than 10.

### 2.2. Experiment on Pervious Hardened Ground

**Figure 1:**
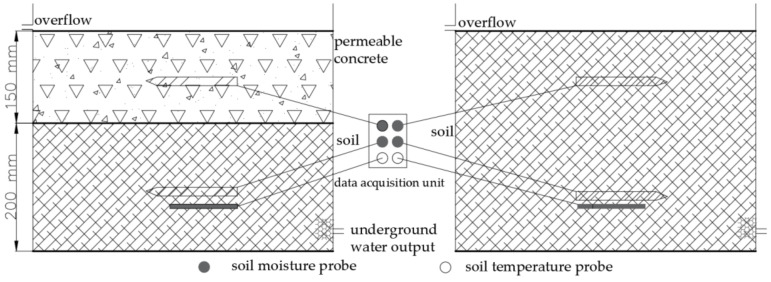
Experimental monitor plan for permeable concrete pavement.

In this study, two organic glass water tanks of 0.5 m × 0.5 m were made. According to the standard atlas of “Urban Road-pervious sidewalk laying” (16MR2004), the experimental model of permeable concrete road was set up, which was installed by professional engineers. HOBO series products were used to monitor and collect soil moisture, concrete moisture and soil temperature data, including the data acquisition unit (HOHO H21-USB), soil moisture probe (S-SMC-M005), and soil temperature probe (HOBO S-TMB-MOO6). For the S-SMC-M005, the volumetric water content range is 0~0.55 m^3^/m^3^, the precision range is ±3.1%, the resolution is 0.0007 m^3^/m^3^ (0.07%), and the effective soil volume is 0.3 L. The range of HOBO S-TMB-MOO6 is −40~100 °C, the precision is ±0.2 °C (0~50 °C), the resolution is 0.03 °C, the response time is <30 s (90%, in flowing water), and < 3 min (90%, 1 m/s in air). The above technical parameters meet the experimental conditions and requirements of this study. The soil moisture monitor plan was shown in Figure 1. The thickness of the permeable concrete structure layer is 150 mm, and the thickness of the lower soil is 200 mm in the experimental group. The soil depth is 350 mm in the control group. The monitoring probe is placed in the middle of the structure layer, so the placement depth of the probes from top to bottom are 75 mm and 250 mm, respectively.

**Figure 2:**
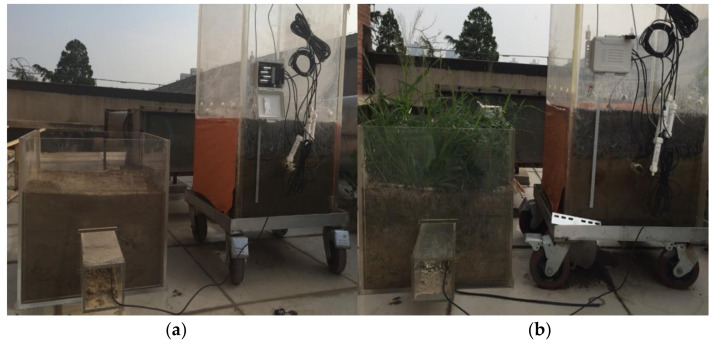
Experimental layout of permeable concrete pavement: (**a**) control group of bare soil; (**b**) control group of nature vegetation.

The change of surface temperature and RH (relative humidity) is recorded by temperature and humidity loggers (WSZY-1) on the surface of concrete. The temperature measurement range is from −40~100 °C with an uncertainty below ±0.5 °C and resolution of 0.1 °C, while the RH measurement range is 0~100% with an uncertainty less than ±3% and resolution of 0.1% RH [31]. In order to avoid the effect of light on permeable concrete and soil evaporation, orange wallpaper was pasted on the water tank wall to shade the light. The site of the experiment is the open-air platform on the fourth floor of the new water conservancy building of Tsinghua University. Two kinds of control conditions were set up in the experiment, one was bare soil surface and the other was natural vegetation surface, and the experimental arrangement was shown in Figure 2. The experimental time of bare soil surface control was 8 May–13 June 2018, and that of natural plant control was from 14 June to 14 July 2018.

### 2.3. Calculation Model

The intercepted rain water is the main source of evaporation on the impervious hardened surfaces. If the maximum water holding depth of the impervious surface is recorded as *H*, when the sum of rainfall depth (*P*) and current water depth (*S*) is less than *H*, the actual evaporation amount (*E_i_*) is equal to the sum of rainfall and current water quantity (*P* + *S*). Conversely, evaporation is equal to the maximum water holding depth *H*. This relationship is expressed in Equation (2).

(2) $$\begin{array}{r} P+S<H,\;E_{i}=P+S\\ P+S\geq H,\;E_{i}=H \end{array}\}$$

On an annual scale, evaporation from impervious ground is equal to accumulated rainfall interception. There is an initial loss value (*H*_0_) for daily rainfall, when the rainfall is less than this value, the rainfall evaporates completely, and when the rainfall is greater than this value, the daily evaporation force is equal to this value (*H*_0_)

(3) $$E_{H}=P_{H}+H_{0}\times N_{H}$$

where *E_H_* is the annual evaporation on the impervious surface, *P_H_* is the sum of the daily rainfall that less than *H*_0_, *N_H_* is the number of days that daily rainfall was greater than *H*_0_.

Road sprinkling is a common municipal public measure to reduce dust and improve the quality of urban environment. Since the road is impervious, all the water that is artificially sprinkled on the road evaporates into the air, so the evaporation of the artificial sprinkling can be calculated by statistical method.

(4) $$W_{A}=1000\times H_{A}\times \left(365-d_{frost}-d_{rain1}\right)\times A_{r}$$

where *W_A_* is the total water content of artificial sprinkling on the imperious road, m^3^; *H_A_* is the depth of artificial sprinkling water, mm (Combined with experimental results and investigation results, the *H_A_* = 0.65 mm in this study); *d_frost_* is the number of frost days; *d_rain_* is the number of raining days; *A_r_* is the area of artificial sprinkler roads, km^2^. In this study, the days number of one year is 365.

Where *E_A_* is the evaporation intensity of artificial sprinkling on the built-up areas, mm; *A_IH_* is the area of imperious hardened surface in cities, km^2^.

(5) $$E_{A}=\frac{W_{A}}{1000\times A_{IH}}$$

Pervious hardened surfaces can penetrate rain water through structural pores and store water in these pores. Their pore structure can connect the lower soil and the upper air and keep the continuous evaporation of the lower soil during the non-rainfall period. The actual water holding capacity is marked as *S_a_*, the rainfall intensity is marked as *p*, the permeability of the pervious layer is recorded as *k*, the water holding capacity of the pervious layer is *S_c_*, the initial water content is *S*_0_, *t* is the duration of rainfall, the rainfall is *P*, and *R* is the runoff on the pervious hardened surface. The relationships between these variables are expressed as follows under different rainfall intensity and rainfall amount.

(6) $$\begin{array}{c} \mathrm{if}\;p<k,\;P<S_{c}-S_{0},\;\mathrm{then}\;S_{a}=S_{0}+P\\ \mathrm{if}\;p<k,\;P>S_{c}-S_{0},\;\mathrm{then}\;S_{a}=S_{c},\;R=P-S_{c}\\ \mathrm{if}\;p>k,\;P>S_{c}-S_{0},\;\mathrm{then}\;S_{a}=S_{c},\;R=P-S_{c}\\ \mathrm{if}\;p>k,\;P<S_{c}-S_{0},\;\mathrm{then}\;S_{a}=S_{0}+kt,\;R=\left(p-k\right)t \end{array}\}$$

## 3. Results

**Figure 3:**
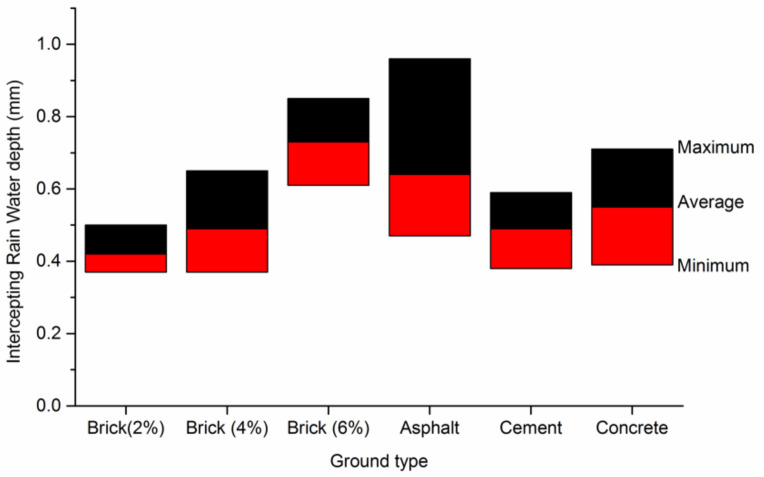
Experimental results of typical impervious surfaces interception.

The monitoring results of typical impervious surfaces interception were shown in Figure 3 through the histogram. According to the experimental monitoring results, the water holding depths of brick, asphalt, cement, and concrete ground were distributed between 0.4–1 mm, and the average value is nearly 0.56 mm, which was close to the depth of artificial sprinkling on roads. The depth of water interception of asphalt pavement was greater than that of other types of impervious pavement. The water holding depth of concrete pavement was slightly higher than that of cement pavement. These differences were mainly due to the different roughness of impervious surfaces with different materials. Without considering the water absorption performance of the material, the ground surface with large roughness has strong intercepting rain water ability, and also has large evaporating strength. For brick pavements, the greater water interception depth happened on the larger gap ratio pavements, because the gap had a certain groove depth and water retention capacity.

**Figure 4:**
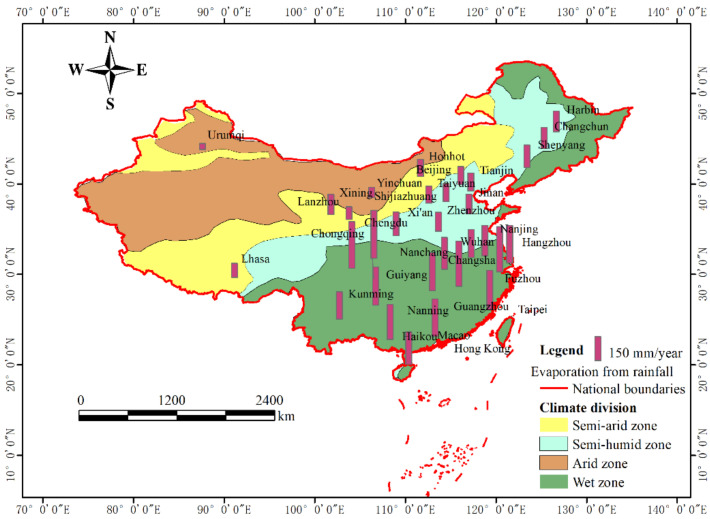
Evaporation intensity of impervious surfaces from rainfall in provincial capital cities in China in 2015.

Based on formula (4) and measured rainfall data in 2015 (Table 1), the evaporation intensity of impervious surfaces in 31 provincial capital cities in China was calculated. Due to the lack of measured meteorological data, there was no calculation of hardened surfaces evaporation in the three cities of Taipei, Macao and Hong Kong. Figure 4 showed the intensity of the evaporation of rainwater intercepted by the impervious surfaces in capital cities in 2015. Based on the experimental data and referring to the recommended value of “Technical Guide for Sponge City Construction in China”, The initial loss value was 2 mm when it was upscaled to regional or larger scales (*H*_0_ = 2) in this study.

The land in China is divided into four geographical regions according to annual rainfall: humid region (annual rainfall is more than 800 mm), semi-humid region (annual rainfall is between 400–800 mm), semi-arid region (annual rainfall is between 200–400 mm), and arid region (annual rainfall is less than 200 mm) [32]. The evaporation intensity of impervious surfaces in cities in humid areas was much higher than that in arid and semi-arid areas. The smallest evaporation intensity city in 2015 was Urumqi, with only 37.3 mm, and the largest value was 298 mm, which happened in Chengdu. The results showed that more than half of the cities have evaporation intensity from rainfall on impervious surfaces above 150 mm in one year, and these cities were mainly distributed in wet areas.

**Figure 5:**
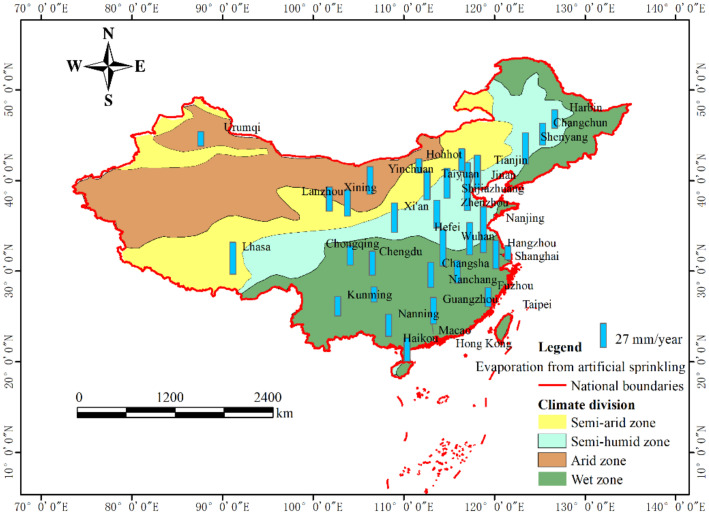
Evaporation intensity of impervious surfaces from artificial sprinkling on built-up areas in provincial capital cities in China in 2015.

According to formula (4) and (5) and rainfall data in Table 1, the evaporation intensity from artificial sprinkling on imperious hardened surfaces of each capital city in 2015 was calculated, and the results were shown in Figure 5. The results showed that more than half of the cities have evaporation intensity from artificial sprinklers on impervious surfaces above 27 mm in one year. These cities were mainly distributed in arid and semi-arid areas. Other cities with relatively low levels of evaporation intensity from artificial sprinkling on impervious ground include four cities in northern China and several cities in wet areas with adequate rainfall. Compared with the evaporation intensity from the rainfall interception, the intensity distribution of artificial sprinkling on impervious surfaces in provincial capital cities were relatively uniform.

**Figure 6:**
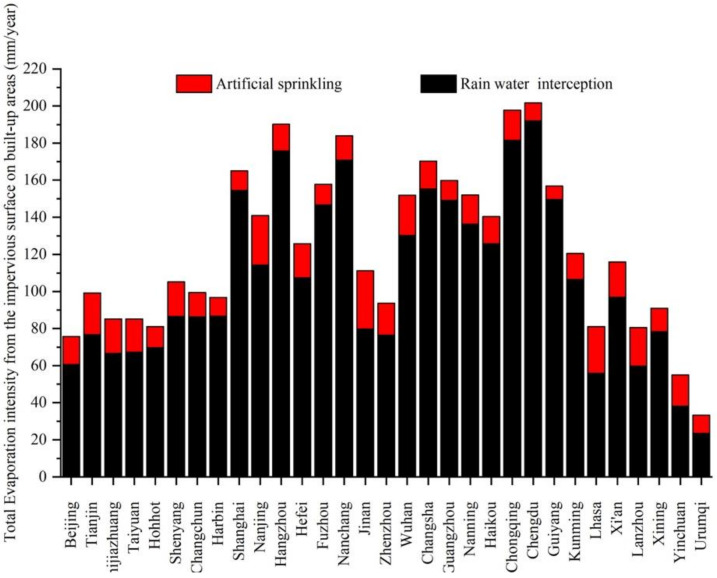
Total evaporation intensity from the impervious hardened surfaces on the built-up area in each capital city.

Total evaporation from impervious surfaces equals the sum of evaporation from rainfall and from artificial sprinkling. In order to analyze the total evaporation intensity of impervious surfaces on the urban built-up areas, the total water volume of evaporation on hardened surfaces from rainfall and artificial sprinklers was evenly distributed on the urban built-up area in each city. The evaporation intensity of the urban impervious surfaces on built-up areas was calculated, and the results were shown in Figure 6. The results showed that interception of rainwater on impervious surfaces accounted for more than 70% of total evaporation from impervious surfaces.

**Figure 7:**
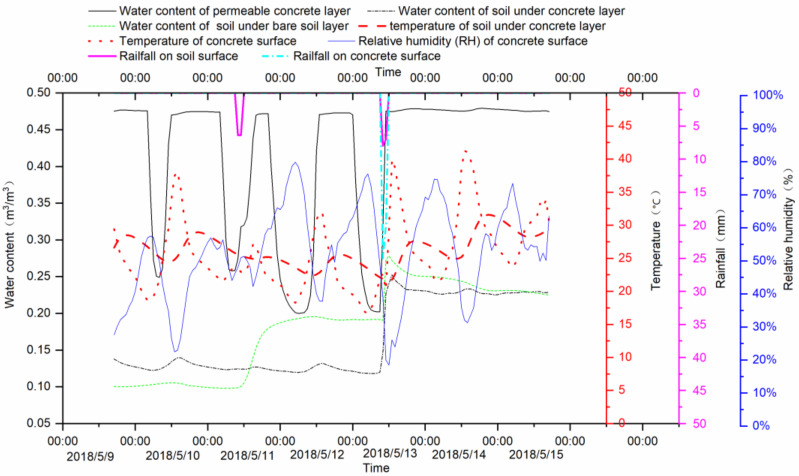
Monitoring results of permeable concrete pavement in the comparison of bare soil surface.

Figure 7 showed the monitoring results of soil moisture change of the experimental group (permeable concrete pavement) and control group (bare soil surface). The experimental monitoring time was 9–15 May 2018. During this period, the control group was subjected to two artificial precipitations, which were 11 May and 13 May, respectively. The experimental group was carried out an artificial precipitation on May 13. This artificial precipitation caused water to accumulate on the surface of the permeable concrete, that is, the water in the permeable concrete layer was saturated on 13 May. Water content of soil under concrete layer and bare soil layer and water content of permeable concrete layer were also measured. When precipitation occurs, the values of three monitored soil moisture were rising rapidly. The water content in the permeable concrete layer rose from the afternoon to the maximum water content, and continued to fall after midnight, thus repeatedly showing periodic change. The soil water content under the permeable concrete and the soil water content in the bare soil showed fluctuations during the non-rainfall period. The fluctuations were reflected in the daily change process, which means, starting to rise every morning, reaching the peak around 3 p.m. Its rise time was ahead of the rise time in permeable concrete. The relative humidity (RH) of the concrete layer surface also changed periodically, but its peak lagged behind the peak of soil moisture in the concrete layer. The temperature in the surface of the concrete layer and the soil in the lower layer also showed periodic changes. The peak of the surface temperature appeared at 12:00 a.m., and the peak of the lower soil temperature appeared at 0:00 a.m.

**Figure 8:**
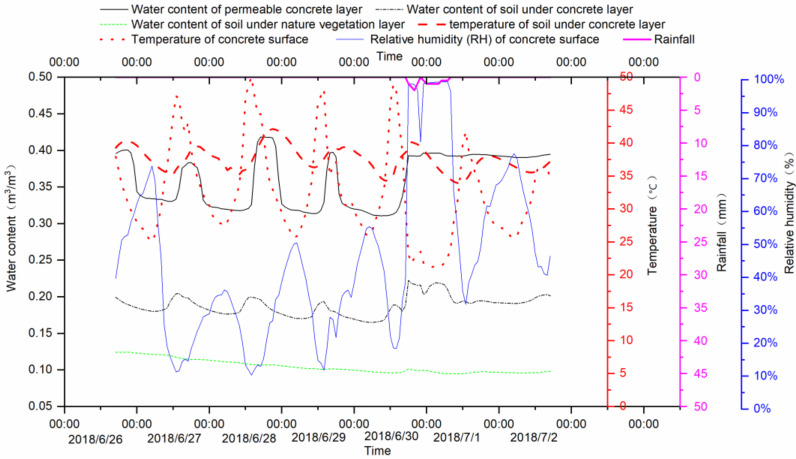
Monitoring results of permeable concrete pavement in the comparison of native vegetation surface.

The Figure 8 showed the monitoring results in the comparison of native vegetation surface. The vegetation was *setaria viridis*, which was naturally grown vegetation in this study (Figure 2b). The monitoring time was between 26 June and 3 July, and a natural rainfall occurred. When rainfall occurred on 1 July, the moisture content of the permeable concrete and the underlying soil rose rapidly and visibly, but the soil moisture under the natural vegetation layer changed by a smaller magnitude. The results showed that the soil moisture in the lower layer of natural vegetation was decreasing smoothly, while the soil moisture in the permeable concrete had diurnal fluctuations. Water content of permeable concrete layer was showing periodic changes during non-rainfall periods, and the trough was gradually declining. The water content of the concrete layer had been in the peak for a few days after the rain. The RH of the concrete surface reached peak during natural rainfall, and the value nearly approached 100%. The temperature change characteristics of the concrete surface and under soil layer were the same as in Figure 7.

## 4. Discussion

The evaporation of impervious hardened surfaces comes from the interception of rain water and artificial sprinkling (suitable for some roads with artificial sprinkling) on the surface. The rainwater interception capacity of impervious surfaces primarily determines the magnitude of surface evaporation. The data are the reference basis for artificial sprinkling on city roads, and this is also the base to calculate the evaporation from artificial sprinkling on the impervious road. Due to limited water storage capacity of impervious surfaces, the evaporation of impervious surfaces is a water-constrained type of evaporation, and further results showed that the number of days of rainfall is the key factor affecting annual evaporation. According to the annual evaporation results in Figure 4, the larger evaporation happened in cities with more days of rainfall, which are usually distributed in wet areas.

Unlike the evaporation of natural rainfall, the larger evaporation of hardened surfaces caused by artificial sprinklers happened in cities with less rainfall days and frost days. In mid-latitude cities with less rainfall, such as Beijing, Tianjin, Taiyuan, Shijiazhuang, Jinan and Zhengzhou, the evaporation from artificial sprinklers is higher than other cities, because they are located in relatively arid areas, with fewer frost days.

Further analysis in Figure 6 found that the evaporation on impervious surfaces from rainfall interception is 10–22 times that from artificial sprinkling in cities in humid areas, while this ratio interval is 3–10 times in cities in arid and semi-arid areas. In order to analyze the effect of evaporation from impervious hardened surfaces on urban evaporation, five cities’ urban evapotranspiration values were collected from the research literature. The five cities are Beijing, Shijiazhuang, Shanghai, Guangzhou and Chengdu. Their average annual urban evapotranspiration were 407 mm, 529 mm, 751 mm, 767 mm, and 730 mm, which were by traditional methods that ignored evaporation from the impervious surfaces. The proportion of impervious surfaces evaporation in traditional urban evapotranspiration is 18.34%in Beijing, 16.37% in Shijiazhuang, 21.97% in Shanghai, 21.90% in Guangzhou, and 28.68% in Chengdu, respectively.

From the monitoring results of Figure 7 and Figure 8, the variation of water content in permeable concrete layers shows a fluctuating pattern. Without new rainfall, the value of the trough is gradually decreasing, indicating that evaporation is continuing to occur, and the period of wave crest and trough variation is one day. The reason for the fluctuating change is that the soil moisture in the lower layer evaporates through the monitoring point. Evaporation is affected by meteorological factors such as surface temperature, humidity, sensible heat and so on. These meteorological elements on the surface of permeable concrete fluctuate periodically in the results of experimental monitoring. This is also the reason for the periodic fluctuation of soil water evaporation in the sub-layer of permeable concrete.

In the control group of bare soil and natural vegetation, the change of soil moisture is close to smooth, which is different from the periodic fluctuation of the same depth in the experimental group. When high intensity heavy rain occurs, the increase of soil moisture in the lower layer of permeable concrete is lower than that in bare soil control scenario. However, when low intensity small rainfall occurs, the increase of soil moisture in the lower layer of permeable concrete is much higher than that in bare soil and natural vegetation conditions.

## 5. Conclusions

Urban hardened surfaces can be divided into impervious hardened surfaces and pervious hardened surfaces. Impervious hardened surfaces will intercept rain water, and this part rain water all evaporates into air. For some roads, there will be municipal artificial sprinkling, and this part of the water also contributes to urban evaporation. The evaporation of impervious hardened surfaces is water constrained evaporation, which means the evaporation happens only when there is water on the impervious surfaces, so this evaporation is intermittent. The annual evaporation of urban impervious surfaces from the interception of rain water is closely related to number of rainfall days. Based on the results of evaporation of hardened surfaces in 31 provincial capital cities in China in 2015, it can be concluded that the distribution intensity of evaporation of hardened surfaces in the built-up area is 40–210 mm. The evaporation of impervious pervious hardened surfaces accounts for 16–29% in the urban evapotranspiration calculated by the traditional methods in five capital cities, which is non-negligible part in the evaporation calculation in urban built-up areas.

Considering that the proportion of pervious hardened surfaces in urban areas is particularly small (negligible compared to impervious hardened surfaces) in China’s cities in 2015, the evaporation of water-pervious hardened surfaces is not taken into account in this study. Through experimental monitoring, we compared the evaporation characteristics of subsoil in pervious hardened surfaces with the bare soil and natural vegetation surface. The results found that the pervious hardened surfaces can penetrate water and connect the lower soil to the surface atmosphere [33]. The evaporation of pervious hardened surfaces is continuous compared to impervious surfaces. The soil moisture in the sub-layer of permeable concrete decreases in a wave-like manner during the absence of rainfall, while the soil moisture under the layer of bare soil or natural vegetation decreases smoothly at the same depth.

The authors concluded that evaporation of the impervious hardened surfaces is intermittent, while evaporating evaporation of the pervious hardened surface is continuous. When there is water on impervious surfaces, the evaporation happens, otherwise there’s no evaporation. For the pervious surfaces, the rainfall can seep, and water from the underlying soil can also pass through the hardened layer and evaporate into the air. The amount of evaporation of urban hardened surfaces occupies a certain proportion between 16–29% of the total amount of evaporation in the built-up areas. The evaporation of hardened surfaces is also an important link of urban hydrological cycle and an important flux of urban water balance.

## Figures and Tables

**Table 1:** Rainfall related data in 31 provincial capitals of China in 2015.

Cities	Rainfall in 2015 (mm)	*d_rain_* (d)	*N_H_* (d)	*d_frost_* (d)	Impervious Area Rate (%)
Beijing	458.6	77	45	5	54.21%
Tianjin	574.2	79	44	5	67.92%
Shijiazhuang	481.4	74	44	4	59.81%
Taiyuan	403.6	79	35	2	63.93%
Hohhot	361.9	77	38	94	66.00%
Shenyang	573.2	93	56	89	61.67%
Changchun	530.5	99	50	117	65.88%
Harbin	420.1	105	49	126	67.19%
Shanghai	1648.8	152	96	0	66.00%
Nanjing	1765.6	124	75	0	60.53%
Hangzhou	2131.9	169	121	0	62.56%
Hefei	1258.2	128	72	0	61.75%
Fuzhou	1778.2	166	98	0	59.92%
Nanchang	2204.7	173	115	0	61.10%
Jinan	713.8	75	54	3	65.00%
Zhengzhou	689.1	86	46	1	64.68%
Wuhan	1432.8	138	79	0	65.81%
Changsha	1538.3	162	100	0	66.10%
Guangzhou	2471.9	248	101	0	63.94%
Nanning	1222.3	156	85	0	63.02%
Haikou	1673.2	135	84	0	61.90%
Chongqing	1416.7	122	84	0	62.62%
Chengdu	1388.6	211	112	0	64.43%
Guiyang	1430.8	193	83	0	62.83%
Kunming	1216.1	122	69	0	62.75%
Lhasa	326.1	64	32	2	64.02%
Xi’an	578.9	103	53	0	65.72%
Lanzhou	266.7	60	25	4	75.86%
Xining	306.2	111	43	15	62.53%
Yinchuan	227.1	53	21	15	59.10%
Urumqi	105.7	33	12	96	63.00%

## Data Availability

Data sharing not applicable.
